# *Staphylococcus aureus* Keratitis: A Review of Hospital Cases

**DOI:** 10.1371/journal.pone.0080119

**Published:** 2013-11-11

**Authors:** Sherine Jue Ong, Yhu-Chering Huang, Hsin-Yuan Tan, David H. K. Ma, Hsin-Chiung Lin, Lung-Kun Yeh, Phil Y. F. Chen, Hung-Chi Chen, Chih-Chun Chuang, Chee-Jen Chang, Ching-Hsi Hsiao

**Affiliations:** 1 Department of Ophthalmology, Chang Gung Memorial Hospital, Linkou, Taiwan; 2 Division of Pediatric Infectious Diseases, Department of Pediatrics, Chang Gung Memorial Hospital, Linkou, Taiwan; 3 College of Medicine, Chang Gung University, Taoyuan, Taiwan; 4 Department of Ophthalmology, Yuan-Sheng Hospital, Changhua, Taiwan; 5 Department of Ophthalmology, Changhua Christian Hospital, Changhua, Taiwan; 6 Graduate Institute of Clinical Medical Science, Chang Gung University, Taoyuan, Taiwan; 7 Clinical Informatics and Medical Statistics Research Center, Chang Gung University, Taoyuan, Taiwan; UC Berkeley, United States of America

## Abstract

**Background:**

Methicillin-resistant *Staphylococcus aureus* (MRSA) infection is an important public health issue. The study aimed to characterize the patient demographics, clinical features, antibiotic susceptibility, and clinical outcomes of keratitis caused by *S. aureus*, and to make a comparison between MRSA and methicillin-sensitive *S. aureus* (MSSA) isolates.

**Methodology/Principal findings:**

Patients (n = 59) with culture-proven *S. aureus* keratitis treated in Chang Gung Memorial Hospital between January 1, 2006, and December 31, 2010, were included in our study. Patients' demographic and clinical data were retrospectively reviewed. Twenty-six MRSA (44%) and 33 MSSA (56%) isolates were collected. The MRSA keratitis was significantly more common among the patients with healthcare exposure (*P* = 0.038), but 46.2% (12/26) of patients with MRSA keratitis were considered to have community-associated infections. All isolates were susceptible to vancomycin. MRSA isolates were significantly more resistant to clindamycin, erythromycin, and sulfamethoxazole/trimethoprim. Ocular surface disease was a significant risk factor for MRSA keratitis (*P* = 0.011). Visual outcome did not differ significantly between the MRSA and MSSA groups. However, age (B = 0.01, *P* = 0.035, 95% confidence interval [CI]: 0.001–0.019) and visual acuity at presentation (B = 0.749, *P*<0.001, 95% CI: 0.573–0.926) were significantly correlated with visual outcome.

**Conclusions/Significance:**

Ocular surface disease is an important predisposing factor for *S. aureus* keratitis, especially for MRSA infections. Advanced age and poor visual acuity at presentation are important prognostic indicators for poor visual outcome in *S. aureus* keratitis. Oxacillin resistance may not be a significant prognostic indicator.

## Introduction

*Staphylococcus aureus* is one of the most important pathogens in bacterial keratitis, a vision-threatening disease. [1], [2] Although the incidence of *S. aureus* keratitis varies worldwide, the increasing trend of resistance to certain antibiotics makes this condition an important global healthcare issue. [3]–[5]

Methicillin-resistant *S. aureus* (MRSA) is a term used to describe strains of *S. aureus* that are resistant to all β-lactam antibiotics. The emergence of MRSA strains is clinically relevant because their resistance to multiple antibiotics limits treatment options for MRSA infection. Formerly considered a nosocomial pathogen, MRSA has reportedly increased in prevalence among otherwise healthy patients without identified risk factors. These infections are described as community-associated MRSA (CA-MRSA) infections. In Western countries, the most common manifestations of ocular MRSA infections are conjunctivitis or lid disorders, [6]–[8] whereas keratitis was the most common ocular diagnosis in our previous study in Taiwan, accounting for 36.1% of the MRSA ocular infections. [9] Previous studies of MRSA keratitis have generally been limited to case reports and small case series. [10]–[15] The scope of our previous studies on ocular MRSA infections was primarily focused on epidemiology and included a broad spectrum of diseases, so we did not intend to analyze clinical features and outcomes. [9], [16]

Herein, we performed a 5-year retrospective study of *S. aureus* keratitis in Chang Gung Memorial Hospital (CGMH), a 3000-bed tertiary referral hospital in Taiwan. We compared the clinical characteristics, predisposing factors, antibiotic susceptibility, treatment modalities, and visual outcome of patients with MRSA keratitis to those caused by methicillin-sensitive *S. aureus* (MSSA)

## Methods

### Ethics

Our study adhered to the guidelines of the Declaration of Helsinki, and was approved by the Institutional Review Board of CGMH, which granted a waiver of consent because patient anonymity was maintained by the data source.

### Participants and procedures

We queried the computer database from the microbiology laboratory in CGMH and reviewed the corresponding medical records to identify patients with *S. aureus* keratitis who were treated between January 1, 2006, and December 31, 2010. Both inpatients and outpatients were included. Data collected included demographic information, medical and ocular history, presenting signs and symptoms, systemic and local predisposing factors, presenting visual acuity (VA), antibiotic susceptibility, treatment, length of follow up, and final VA. The size and location of corneal infiltrates and the presence of hypopyon were also documented.

We determined the susceptibility of bacterial isolates to clindamycin, erythromycin, cefoxitin, penicillin, trimethoprim/sulfamethoxazole, teicoplanin, and vancomycin using the disk diffusion method based on the standards for antimicrobial susceptibility testing established by the Clinical and Laboratory Standard Institute (CLSI). The isolates were stored for additional testing for susceptibility to fluoroquinolones (ciprofloxacin, levofloxacin, and moxifloxacin), at a later date in 2010. We used cefoxitin to test for β-lactam antibiotic resistance.

We defined healthcare-associated MRSA (HA-MRSA) and CA-MRSA according to the definitions proposed by Naimi et al. [17] Patients meeting one or more of the following criteria were considered HA-MRSA cases: (1) a MRSA infection identified within 48 hours after admission to a hospital; (2) a history of hospitalization, surgery, dialysis, or residence in a long-term care facility within one year of a MRSA culture date; (3) a permanent indwelling catheter or percutaneous medical device present at the time of culture; or (4) a known positive culture for MRSA before the beginning of the study period. Cases meeting none of these criteria were defined as CA-MRSA infection.

To treat *S. aureus* keratitis, empiric or fortified antibiotics were administered hourly. The standard fortified antibiotics consisted of topical amikacin (25 mg/ml), cefazolin sodium (25 mg/ml), or vancomycin (25 mg/ml); the commercially available antibiotics used were topical fluoroquinolones (ciprofloxacin 0.3% or levofloxacin 0.5%). The antibiotic treatment regimens were adjusted subsequently according to the culture results, the antibiotic susceptibility, and the clinical response. Surgical interventions, including amniotic membrane transplantation, tarsorrhaphy, patch graft, or therapeutic penetrating keratoplasty, were performed as needed. The healing time was recorded after infiltration had subsided and the epithelial defect had healed. The VA was recorded at >2 months after the keratitis had subsided and stabilized, and Snellen VA values were converted into logMAR units for statistical analysis. VA of counting fingers, hand movements, light perception, and no light perception (NLP) were recorded as logMAR units as described previously. [18]

The Genotyping analyses, including pulsed-field gel electrophoresis (PFGE) typing, SCC*mec* elements and the detection of Panton-Valentine leukocidin (PVL) genes, were performed in available MRSA isolates. PFGE was used to fingerprint the MRSA clinical isolates according to the procedure described previously. [19] The criteria proposed by Tenover et al. [20] were employed to analyze the DNA fingerprints generated by PFGE. The SCC*mec* typing was determined by a multiplex polymerase chain reaction (PCR) strategy described previously. [21] Control strains for SCC*mec* types I, II, III, and IVa, kindly provided by Dr K. Hiramatsu, were as follows: type I, NCTC10442; type II, N315; type III, 85/2082; and type IVa, JCSC4744. The PCR amplification of the *lukS-PV* and *lukF-PV* genes encoding PVL components was performed as described previously. [22]

### Statistical analysis

All statistical analyses were performed using the SPSS, Version 17, computer software (IBM, Armonk, NY, USA). A chi-square test or Fisher’s exact test (when the expected value < 5) was used to compare the nominal variables. The Mann-Whitney *U* test was used for continuous variables. Linear stepwise regression was used to determine the factors associated with visual outcome. Statistical significance was defined as *P* < 0.05.

## Results

### Demographics (Table 1)

**Table 1:** Comparison of demographics of keratitis caused by MRSA and MSSA.

Characteristics	MRSA (n = 26)	MSSA (n = 33)	*P* value
Age (years): median (min-max)	55 (2–83)	56 (1–83)	0.743
Gender: M/F	15/11	17/16	0.636
Eye: R/L/B	9/16/1	20/13/0	0.084*
Community associated/Healthcare-associated: n. (%)	12 (46.2)/14 (53.8)	24 (72.7)/9 (27.3)	0.038

* : *P* value obtained by Fisher's Exact Test.

MRSA: methicillin-resistant *Staphylococcus aureus*, MSSA: methicillin-sensitive *Staphylococcus aureus*, M: male, F: female. R: right eye, L: left eye, B: both eyes.

Fifty-nine cases of *S. aureus* keratitis were identified, including 26 (44%) caused by MRSA and 33 (56%) caused by MSSA. No significant difference in sex, age, or laterality was observed between the MRSA and MSSA cases. The rate of healthcare-associated infection was significantly higher in the MRSA group (14/26, 53.8%) than that in the MSSA group (9/33, 27.3%; *P* = 0.038). There was no significant difference in the mean follow-up period between the MRSA (1.65 ± 1.41 years) and MSSA cases (1.57±1.69 years; *P* = 0.388).

### Clinical findings (Table 2)

**Table 2:** Clinical findings of MRSA and MSSA keratitis.

Clinical findings	MRSA (n = 26) No. (%)	MSSA (n = 33) No. (%)	*P* value
Location			0.01
Central	16 (61.5)	7 (21.2)	
Paracentral	6 (23.1)	13 (39.4)	
Peripheral	4 (15.4)	13 (39.4)	
Infiltration size (mm)			0.133*
Small (<2)	3 (11.5)	12 (36.3)	
Medium (2∼6)	20 (77.0)	18(54.6)	
Large (>6)	3 (11.5)	3 (9.1)	
Hypopyon	5 (19.2)	8 (25.0)	0.6

* Fisher's Exact Test.

MRSA: methicillin-resistant *Staphylococcus aureus*, MSSA: methicillin-sensitive *Staphylococcus aureus*,

The MRSA cases had a significantly higher rate of centrally located corneal ulcer, as defined by the centration of the corneal infiltrate, with central ulcers in 61.5% (16/26) of the MRSA cases, whereas 21.2% (7/33) of the MSSA cases (*P* = 0.01) had centrally located corneal ulcers. No significant difference was observed in the infiltration size or the presence of hypopyon between the MRSA and MSSA groups.

### Predisposing factors (Table 3)

The local and systemic predisposing factors for *S. aureus* keratitis are summarized in Table 3. The most common predisposing factor for both the MRSA and MSSA keratitis was ocular surface disease, accounting for 62.7% (37/59) of all *S. aureus* keratitis. Additionally, patients with ocular surface disease had a significantly greater risk of MRSA keratitis (*P* = 0.011). No significant difference in the other local risk factors, including wearing of contact lenses, trauma, previous ocular surgery, or local use of immunosuppressants/antibiotics, were observed between the MRSA and MSSA groups. Furthermore, no significant difference in systemic risk factors for keratitis, including the presence of underlying comorbidities or the systemic use of immunosuppressants or antibiotics, were observed between the MRSA and MSSA groups.

**Table 3:** Predisposing factors for MRSA and MSSA keratitis.

Predisposing Factors	MRSA (n = 26)	MSSA (n = 33)		*P* value
Local factors, n (%)*				
Conact lens wear	4 (15.4)	5 (15.2)		1†
Trauma	1 (3.8)	3 (9.1)		0.623†
Ocular surface disease	21 (80.8)	16 (48.5)		0.011
Previous ocular surgery	10 (38.5)	13 (39.4)		0.942
Usage of topical antibiotics or immunosuppressant	9 (34.6)	6 (18.2)		0.15
Systemic factors n (%)*				
Systemic comorbidities	17 (65.4)	16 (48.5)		0.194
Immunosuppressant	1 (3.8)	2 (6.1)		1†
Systemic antibiotics	2 (7.7)	3 (9.1)		1†

* : Total is greater than 100% because of some patients with multiple risk factors.

† : *P* value obtained by Fisher's Exact Test.

MRSA: methicillin-resistant *Staphylococcus aureus*, MSSA: methicillin-sensitive *Staphylococcus aureus*.

### Antibiotics susceptibility (Table 4)

**Table 4:** Antibiotics susceptibility of MRSA and MSSA isolates for keratitis.*

Antibiotics	MRSA (n = 26)No. (%)	MSSA (n = 33)No. (%)	*P* value
Clindamycin	0 (0)	31 (93.9)	<0.001
Erythromycin	0 (0)	29 (87.9)	<0.001
Oxacillin	0 (0)	33 (100)	<0.001
Penicillin	0 (0)	4 (12.1)	0.123†
Sulfamethoxazole/trimethoprim	16 (61.5)	33 (100)	<0.001
Teicoplanin	26 (100)	33 (100)	
Vancomycin	26 (100)	33 (100)	
Ciprofloxain‡	5 (62.5)	7 (77.7)	0.437†
Levofloxacin‡	6 (75)	9 (100)	0.206†
Moxifloxain‡	6 (75)	9 (100)	0.206†

* : Intermediate susceptibility regarded as resistant.

† : *P* value obtained by Fisher's Exact Test.

‡ : Eight MRSA and nine MSSA isolated were tested for fluoroquinolones.

MRSA: methicillin-resistant *Staphylococcus aureus*, MSSA: methicillin-sensitive *Staphylococcus aureus*.

The MRSA isolates were significantly more resistant to clindamycin, erythromycin, oxacillin, and sulfamethoxazole/trimethoprim than the MSSA isolates. Sixteen of 26 (61.5%) MRSA isolates were susceptible to sulfamethoxazole/trimethoprim. All the MRSA and MSSA isolates were susceptible to both vancomycin and teicoplanin. Of the eight MRSA and nine MSSA isolates that were available for ciprofloxacin, levofloxacin, and moxifloxacin susceptibility testing, two MRSA isolates were resistant to all three tested fluoroquinolones. One MRSA isolate and two MSSA isolates were resistant to ciprofloxacin, but susceptible to levofloxacin and moxifloxacin.

### Treatment and outcomes (Table 5)

**Table 5:** Treatment and clinical outcome of MRSA and MSSA keratitis.

	MRSA (n = 26)	MSSA (n = 33)	*P* value
Administration of topical antibiotics: n (%)	26 (100)	33 (100)	
Modification of antibiotics: n (%)	17 (65.4)	15 (45.5)	0.057
Surgical intervention: n (%)	9 (34.6)	9 (27.3)	0.543
Admission: n (%)	13 (50)	14 (44.4)	0.922*
Severe complications:† n (%)	4 (15.4)	4 (12.1)	0.722*
Healing time‡ (months)median (mini∼max)	1.2 (0.2∼8.5)	0.83 (0.1∼11.7)	0.348
VA (Log MAR): median (mini∼max)			
At presentation	2.6 (0∼3)	1.65 (0∼3)	0.084
After treatment	2.3 (0∼3.2)	1.7 (0∼3.2)	0.449

* : *P* value obtained by Fisher's Exact Test.

† : Severe complications: corneal perforation and/or endophthalmitis.

‡ : Healing time: defined as the resolution of infiltration and epithelial defect.

MRSA: methicillin-resistant *Staphylococcus aureus*, MSSA: methicillin-sensitive *Staphylococcus aureus*, VA: visual acuity, Log MAR : logarithm of the minimum angle.

All patients were treated with topical antibiotics. A combination of fortified antibiotics (cefazolin sodium 25 mg/ml and amikacin 25 mg/ml) or fluoroquinolone alone (ciprofloxacin 0.3% or levofloxacin 0.5%) was the most common empiric treatment. In 32 cases, the medication regimen was shifted to vancomycin 25 mg/ml after the culture results were obtained, but the rate of modification did not differ significantly between the MRSA and MSSA groups (*P* = 0.057). The difference in choice of first-line treatment of the *S. aureus* keratitis was not significantly associated with a need to modify the therapy (*P* = 0.660 and 0.765 for the MRSA and MSSA groups, respectively).

Nine patients in each group required surgical intervention. Five patients were refractory to medical treatment, four of which (two from each group) underwent therapeutic penetrating keratoplasty or patch graft with glycerol-preserved cornea, whereas one patient who presented with NLP resulting from severe MRSA keratitis received evisceration. Four patients received amniotic membrane transplantation or tarsorrhaphy to promote reepithelialization. No significant difference in the rate of surgical intervention, admission rate, the rate of severe complications (including corneal perforation and endophthalmitis), or healing time were observed between the MRSA and MSSA cases.

The VA was recorded for all patients, except for four patients for whom data were missing and nine patients who were unable to complete the VA evaluation because of severe systemic disease. No significant difference in final visual outcome was observed between the MRSA and MSSA groups. Univariate linear regression analysis revealed that the following eight factors were significantly correlated to poor visual outcome: age (*P* = 0.001), infiltration size (*P* = 0.008 and 0.013), poor initial VA (*P* < 0.001), the presence of severe complications (*P* = 0.012), surgical intervention (*P* = 0.018), previous ocular surgery (*P* = 0.047), ocular surface disease (*P* = 0.036), and systemic disease (*P* = 0.014). The stepwise linear regression analysis, which included these eight factors, confirmed that advanced age (B = 0.01, *P* = 0.035, 95% confidence interval [CI]: 0.001–0.019) and poor initial VA (B = 0.749, *P* < 0.001, 95% CI: 0.573–0.926) were positively correlated with poor visual outcome.

### Genotyping analysis

Eight MRSA isolates were available for genotyping analysis. One of the HA-MRSA isolates (*n* = 2) was characterized as PFGE type F/SCC*mec* II/PVL-negative, and the other was PFGE type A/SCC*mec* IIIA/PVL-negative. These results are consistent with those of the HA-MRSA isolates in our previous study. [23] Five of the six CA-MRSA isolates were characterized as PFGE type C/SCC*mec* IV/PVL-negative and the other was PFGE type D/SCC*mec* V_T_/PVL positive. These CA-MRSA clones shared genetic characteristics that were common to CA-MRSA strains previously indentified in Taiwan. [23].

## Discussion

Our data showed that MRSA and MSSA contributed almost equally to *S. aureus* keratitis and nearly half of MRSA keratitis was community-associated. *S. aureus*, which accounts for approximately 8–22% of all bacterial keratitis, is an important cause of bacterial keratitis. [1], [3], [5], [24], [25] However, studies of the prevalence of MRSA keratitis are scant. Lichtinger et al. [5] reported MRSA present in 1.3% of the *S. aureus* isolates in an 11-year review of microbial keratitis in Canada. The proportion of MRSA keratitis in *S. aureus* keratitis may parallel that of MRSA in ocular *S. aureus* infections, estimates of which vary worldwide from 3% to 53% within a single institution. [6]–[9] The relatively high proportion of MRSA keratitis cases in our current study is consistent with the finding of previous reports of MRSA prevalence at our institution and other hospitals in Taiwan. [9] As we expected, a significantly greater proportion of MRSA keratitis patients were classified as healthcare associated infections than that observed in the MSSA keratitis patients, but CA-MRSA also played a role. Our findings are consistent with those of previous studies that reported an increasing frequency of CA-MRSA isolates in Taiwan and elsewhere. [26], [27] _ENREF_35Because many ophthalmic patients are seen and treated in an outpatient setting, CA-MRSA is likely to be an important source of *S. aureus* keratitis.

Although a significantly greater proportion of the MRSA keratitis patients in our current study had a corneal ulcer that was centrally located, compared with those of the MSSA cases, no significant difference in the clinical manifestations was found between the MRSA and MSSA groups. Shanmuganathan et al. [6] and Freidlin et al. [8] reported that MRSA keratitis was not usually destructive and vision-threatening. Sotozono et al. [12] described 12 cases of keratitis caused by MRSA and methicillin-resistant *S. epidermidis* (MRSE), among which most cases presented as intraepithelial corneal infiltration and superficial keratitis, with only one patient presenting with perforation caused by severe MRSA destructive keratitis. In our current series, most patients in both the MRSA and MSSA groups had medium-sized infiltrates, with four (15.4%) of the MRSA cases and four (12.1%) of the MSSA cases presenting with corneal perforation or endophthalmitis. Our findings reflect more severe complications than those reported in previous studies of MRSA keratitis.

The most common predisposing factor for *S. aureus* keratitis in our series was ocular surface disease, and patients with MRSA keratitis also had a higher frequency of pre-existing ocular surface disease. Previous reports have shown that ocular surface disease is a significant risk factor for MRSA keratitis. [6], [8], [12] *S. aureus* is one of the common flora in the conjunctival sac. Hori et al. [28] found that 1% of preoperative patients carried MRSA on the conjunctiva. Fukuda et al. [29] reported that MRSA can appear as normal conjunctival flora in as many as 10.3% of elderly patients. Kato and Hayasaka [30] studied 628 pre-operative patients, among whom 10 patients (17 eyes) were culture-positive for MRSA or MRSE on the conjunctiva; the risk factors for colonization of MRSA or MRSE included patients older than 80 years of age, carriage of the same bacterial strains in the anterior nares and the throat, nasolacrimal duct obstruction, dry eye and recent hospitalization. In addition, local immunocompromised status could present an opportunity for the colonized MRSA to become pathogenic. In our patients with MRSA keratitis, the ocular surface diseases features included dry eye, exposure keratitis, trichiasis, Stevens-Johnson syndrome, and ocular pemphigoid. Such patients often had compromised integrity of the ocular surface and corneal epithelial defect; they were commonly treated with topical corticosteroids and antibiotics, and they sometimes wore therapeutic contact lenses. All these factors may have predisposed our patients to MRSA corneal infection.

Vancomycin was the most effective agent against the corneal MRSA isolates in the current study. In addition, sulfamethoxazole-trimethoprim retained some degree of activity against the MRSA isolates from our patients. Although the findings are consistent with other reports for ocular MRSA infection, [9], [31], [32] sulfamethoxazole-trimethoprim was less effective in our series than in previous studies. Recent studies have reported an increasing rate of *in vitro* resistance to fluoroquinolones in MRSA ocular isolates. [3], [4], [33] We did not test fluoroquinolones in our microbiological laboratory because they are not included in the recommended list of antibiotics published by the CLSI. Only eight MRSA and nine MSSA isolates in 2010 were stored and available for subsequent fluoroquinolones susceptibility testing; 75% (6/8) of the MRSA isolates were susceptible to levofloxacin and moxifloxacin, although they were less susceptible to the newer fluoroquinolones than were the MSSA isolates (100%). Marangon et al. [4] analyzed the *S. aureus* isolates from keratitis and conjunctivitis in the USA from January 2000 to December 2001. They reported ciprofloxacin and levofloxacin-resistance rates of 11.9% and 4.7%, respectively, for MSSA isolates and 95.7% and 82.1%, respectively, for the MRSA isolates. In addition, the Surveillance Network, which monitors the antimicrobial susceptibility patterns of bacterial pathogens in the USA, reported that, between October 2005 and June 2006, the susceptibility to all fluroquinolones was 79.9% to 81.1% for MSSA isolates and 15.2% for MRSA isolates. [31] Although the sample size may have been too small for drawing reliable conclusions, our results may have differed from these previous reports because of differences in geographic locations, time periods and study populations. Because fluoroquinolones are effective broad-spectrum antibiotics that are commonly used for empiric monotherapy for bacterial keratitis, future studies of the difference in fluoroquinolones susceptibility between MRSA/MSSA isolates and CA-MRSA/HA-MRSA strains are warranted. Serum standards are currently used to interpret the susceptibility of ocular isolates because no specific ocular standards exist. However, the serum standards may not provide an accurate assessment of antibiotic resistance in ocular isolates because the higher antibiotic concentrations of topical instillation may overcome the resistance defined by minimum inhibitory serum concentrations. [34].

Because the rate of MRSA infection is increasing worldwide, we studied the difference in virulence between the MRSA and MSSA isolates. The mortality rates among MRSA bacteremia and infective endocarditis have been shown to be higher than those of MSSA infections. [35], [36] However, we found no statistically significant difference in visual outcome between the MRSA and MSSA cases. In addition, we observed no significant difference in the rate of perforation or endophthalmitis, healing time, or surgical treatment. In a recent study of 32 *S. aureus*-related cases of endophthalmitis, Major et al.[37] found no significant difference in VA outcome between MRSA and MSSA eyes over a 3-month follow-up. Thus, MRSA and MSSA may exhibit similar virulence in ocular infections, but the statistical power of the study was limited by the relatively small sample size.

In our current study, advanced age and poor initial VA, rather than oxacillin resistance, were associated with poor visual outcome in patients with *S. aureus* keratitis. Several risk factors have been reported to predict poor visual outcome in microbial keratitis. Otri et al. [2] conducted a 3-year prospective study of patients with sight-threatening corneal ulcer in the UK and concluded that advanced age, steroid use, and poor VA at presentation were important prognostic indicators. Based on the need for penetrating keratoplasty, Miedziak et al. [38] concluded that advanced age, delay in referral to a corneal specialist, topical steroid treatment, past ocular surgery, poor VA at presentation, large ulcer size, and central location of the ulcer are risk factors for poor outcomes in microbial keratitis. Older patients may have a higher prevalence of ocular morbidities, such as a compromised ocular surface, cataract, or macular degeneration, that may be at least partially responsible for poor visual outcome in microbial keratitis. As expected, the severity of the presenting VA was also significantly related to visual outcome. Combined with those of previous studies, our findings should heighten clinicians' concern for older patients or patients with poor presenting VA, and suggest that such patients should be treated more aggressively to avoid poor visual outcome.

The limitations to our study include the retrospective design and relatively small sample size. Treatment protocols varied among physicians, and there were also inherent flaws associated with using VA as an outcome measure based on assessments made at variable intervals. In addition, we used cefoxitin testing as a surrogate to identify MRSA strains, and we defined CA-MRSA and HA-MRSA based on epidemiological criteria, rather than on genetic characterization. However, the limited molecular results increased our confidence in using epidemiological criteria to classify CA- and HA-MRSA. Furthermore, all our patients came from a referral-based, tertiary-care institution. Thus, the patient selection criteria may have influenced our results. Caution should be exercised in extending our findings to other patient populations.

In conclusion, our 5-year retrospective study found that nearly half of the *S. aureus* keratitis cases at our hospital were caused by MRSA. Although the MRSA strains were significantly more prevalent among the patients with healthcare exposure, 46% of MRSA cases were classified as community associated infections. All the MRSA isolates were susceptible to vancomycin. Although there was no significant difference in visual outcome between the MRSA and MSSA cases, advanced age and poor VA at presentation were significantly associated with poor visual outcome. Our findings provide important information about the clinical profiles of *S. aureus* keratitis, especially for MRSA keratitis, that may help clinicians choose the most appropriate treatment and make more accurate prognoses.

## Acknowledgments

We thank Hsiao-Jung Tseng at the Biostatistical Center for Clinical Research, Chang Gung Memorial Hospital, Taiwan for assistance with the statistical analyses.
